# Editorial: Machine learning approaches for differential diagnosis, prognosis, prevention, and treatment of digestive system disorders

**DOI:** 10.3389/fmolb.2024.1521170

**Published:** 2024-11-13

**Authors:** Xinyan Wu, Xiaomei Zheng, Gang Ye

**Affiliations:** College of Veterinary Medicine, Sichuan Agricultural University, Chengdu, China

**Keywords:** digestive system diseases, machine learning, diagnosis, prognosis, prevention

Digestive System Diseases (DSD) primarily include diseases of the gastrointestinal tract as well as those affecting organs such as the spleen, liver, gallbladder, and pancreas. Clinically, diagnoses are categorized into organic diseases and functional diseases. The pathological mechanisms of DSD are complex, with numerous pathogenic factors, making them significant determinants of life expectancy and quality of life. For example, pancreatic cancer (Yang et al., 2024), colon cancer (Zhang et al., 2022), and ehepatocellular carcinoma (HCC) (Zhang et al., 2023) are the leading causes of death associated with DSD, while cirrhosis and other chronic liver diseases are among the most common DSDs (Zheng et al., 2023).

Machine Learning (ML), as an important technology in the field of artificial intelligence, primarily studies how to learn patterns from large amounts of empirical data to classify and predict unknown data (Chen et al., 2024). By analyzing extensive clinical data, ML can reveal the inherent relationships within the data and generate corresponding predictive models, reflecting the patterns in the diagnosis and treatment of DSD from multiple dimensions, thereby achieving individualized, precise, and digitalized treatment. Additionally, mining feature genes with discriminative capabilities is a key step in elucidating the relationship between genes and DSD, gaining a deeper understanding of disease mechanisms, and improving clinical diagnostic accuracy.

This Research Topic comprises seven original studies, highlighting the applications of ML and deep learning in the investigation of DSD. The included papers cover the use of ML to predict potential biomarkers and their associated mechanisms in DSD, the development of diagnostic differentiation models, and prognosis analysis. This editorial aims to distill the essence of the published research in this field, thereby providing valuable insights and facilitating further research for interested scholars.

Identifying genes related to sample phenotypes, known as feature genes, is a core issue in the analysis of gene expression data for DSD and a challenging aspect of feature selection. The selection of feature genes involves choosing the optimal subset from the existing gene pool, which is crucial for constructing efficient, generalizable classifiers with strong predictive performance. For instance, Zhang et al. Employed ML algorithms to screen feature genes from a dataset of patients with hepatitis B virus-related liver cirrhosis (HBV-LC) and developed both an Artificial Neural Network (ANN) model and a nomogram model. The study demonstrated that models built using feature genes exhibited excellent predictive capabilities, providing important references for the early diagnosis of HBV-LC. Similarly, Zhou et al. Developed a nomogram model for diagnosing HCC using ML, which effectively predicted the early mortality risk in elderly patients with HCC. Furthermore, Qiao et al. utilized methods such as Random Forests (RF), Least absolute shrinkage and selection operator (LASSO) regression, and multivariate Cox regression analysis to construct and validate a prognostic nomogram model. This model accurately predicted 1-year, 3-year, and 5-year recurrence-free survival, offering new perspectives for identifying high-risk populations. Matboli et al. developed various supervised ML models, including Logistic Regression (LR), k-Nearest Neighbors (kNN), Neural Networks (NN), Support Vector Machines (SVM), and RF, to predict treatment responses in HCC. These models integrated a comprehensive set of molecular, biochemical, and immunohistochemical features to evaluate the responses to three drugs: pantoprazole, cyanidin-3-glucoside (Cyan), and hesperidin. The results indicated that the NN model achieved the highest prediction accuracy, and models combining molecular and biochemical features demonstrated outstanding predictive performance. Additionally, the study identified seven molecular features, seven biochemical features, and one immunohistochemical feature as promising biomarkers for treatment response.

Zhang et al. integrated whole sequencing data to construct features associated with regulatory T cells (Tregs), referred to as TAS. They discovered that patients with esophageal squamous cell carcinoma (ESCC) exhibiting high TAS had significantly improved prognoses. Additionally, high TAS samples showed enhanced immune infiltration and increased expression of immune checkpoint markers. The model was validated using the IMvigor210 dataset, demonstrating its effectiveness in predicting prognosis and immunotherapy responsiveness. This indicates that patients with high TAS derive more substantial therapeutic benefits from immune interventions.

Similarly, Shi et al. investigated sphingolipid genes related to the prognosis of pancreatic adenocarcinoma (PAAD), identifying 32 sphingolipid genes that significantly impact overall survival dynamics. These genes formed the conceptual framework of the prognostic model, which, after careful selection, consisted of 10 genes. This innovative risk model, based on the complexity of sphingolipid-related genes, deepened our understanding of PAAD and provided clinicians with a powerful tool for prognosis assessment. Wang et al. employed 10 ML algorithms combined with 81 different configurations to integrate frameworks and establish the optimal prognostic features related to T cell differentiation. These features were validated through multi-cohort transcriptomic analyses. The study identified LDHA as a key marker gene involved in the progression of T cells from non-alcoholic fatty liver disease (NAFLD) cirrhosis to HCC.

In summary, the aforementioned studies highlight the application and dynamic advancements of ML in the research of DSD.
